# Case Report: Postoperative Recurrence of Adrenal Epithelioid Angiosarcoma Achieved Complete Response by Combination Chemotherapy With Liposomal Doxorubicin and Paclitaxel

**DOI:** 10.3389/fonc.2021.791121

**Published:** 2021-12-16

**Authors:** Hangping Wei, Jie Mao, Yandan Wu, Qinfei Zhou

**Affiliations:** ^1^ Department of Medical Oncology, Dongyang Hospital Affiliated to Wenzhou Medical University, Dongyang, China; ^2^ Department of Radiology, Dongyang Hospital Affiliated to Wenzhou Medical University, Dongyang, China; ^3^ Department of Gynaecology and Obstetrics, Dongyang Hospital Affiliated to Wenzhou Medical University, Dongyang, China; ^4^ Department of Rare and Head and Neck Oncology, Cancer Hospital of the University of Chinese Academy of Sciences (Zhejiang Cancer Hospital), Hangzhou, China; ^5^ Institute of Cancer and Basic Medicine (IBMC), Chinese Academy of Sciences, Hangzhou, China

**Keywords:** epithelioid angiosarcoma, adrenal gland, liposomal doxorubicin, paclitaxel, combination chemotherapy

## Abstract

**Background:**

Primary adrenal epithelioid angiosarcoma is an extremely rare cancer with a poor prognosis. Because of the rarity of this disease, treatment options have not been well-studied.

**Case presentation:**

A 51-year-old man was admitted to Zhejiang Cancer Hospital, diagnosed with a recurrence of adrenal epithelioid angiosarcoma. He had undergone a surgical resection seven months earlier. Combination chemotherapy with liposomal doxorubicin and paclitaxel was administered. After two cycles of chemotherapy, his pain was relieved. Computed tomography (CT) suggested that the soft tissue tumour lesions in the surgical area had disappeared, mediastinal and mediastinal-hilar lymph nodes were significantly reduced or had disappeared, and the patient had achieved a partial response (PR). CT after six cycles of chemotherapy indicated that the patient had achieved a complete response (CR).

**Conclusion:**

Combination chemotherapy with liposomal doxorubicin and paclitaxel may be a preferred therapy for recurrent or advanced adrenal epithelioid angiosarcoma.

## Introduction

Angiosarcoma is a rare, highly malignant, soft tissue tumour that originates from vascular endothelial cells and accounts for less than 1% of all soft tissue sarcomas (1, 2). It localises mainly in the deep, soft tissues of the extremities, although visceral sites, including the lung, breast, liver, and bone, have been reported (2). Epithelioid angiosarcoma is a unique morphologic subtype of angiosarcoma that is histologically mostly composed of epithelioid cells and is highly aggressive (3). Primary angiosarcoma of the adrenal gland is extremely rare and is usually epithelioid (4). Up to now, about 40 cases of adrenal epithelioid angiosarcoma have been reported worldwide, and the aetiology remains unknown. At present, chronic lymphoedema, familial angiodysplasia, prior anabolic steroid therapy, and exposure to environmental toxins have been identified as predisposing factors (2). Because of the rarity of this disease, treatment options have not been well-studied. Furthermore, epithelioid angiosarcomas are prone to metastasis and have an unfavourable prognosis, even in cases where surgical removal of the tumour is possible (2, 5). To our knowledge, there are no cases of adrenal epithelioid angiosarcomas with excellent treatment responses. Herein, we report on a case of adrenal epithelioid angiosarcoma recurrence after surgery that achieved complete response (CR) by means of combination chemotherapy with liposomal doxorubicin and paclitaxel.

## Case Presentation

**Figure 1:**
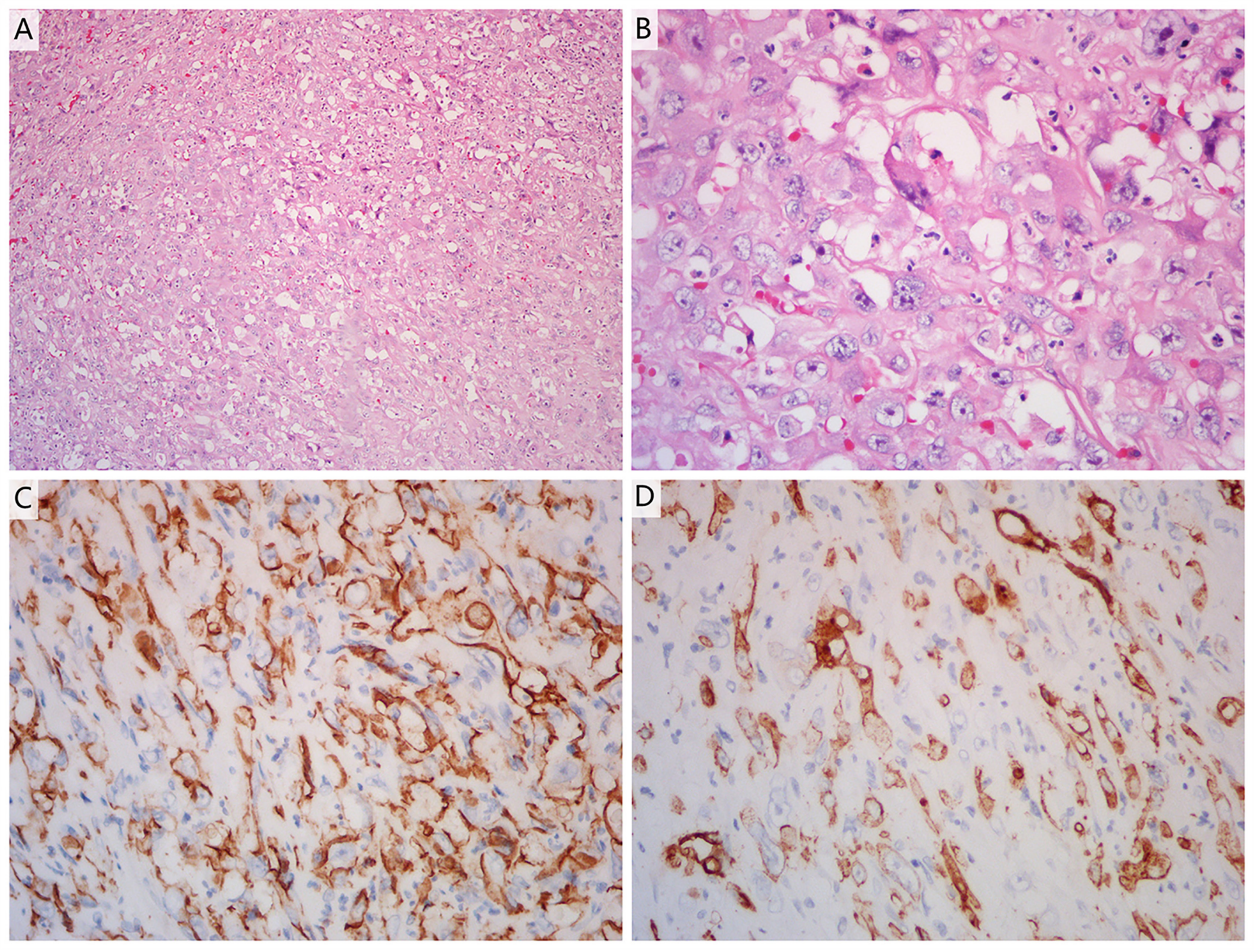
**(A, B)** Haematoxylin and eosin staining of a tumour section (**A**: ×10 magnification; **B**: ×40 magnification). The pathological diagnosis was adrenal angiogenic malignant tumour with massive necrosis. **(C, D)** Immunohistochemistry showed positive reactivity for CD31 **(C)** and CD34 **(D)** in the tumour cells, indicating that they were of endothelial origin (×40 magnification).

**Figure 2:**
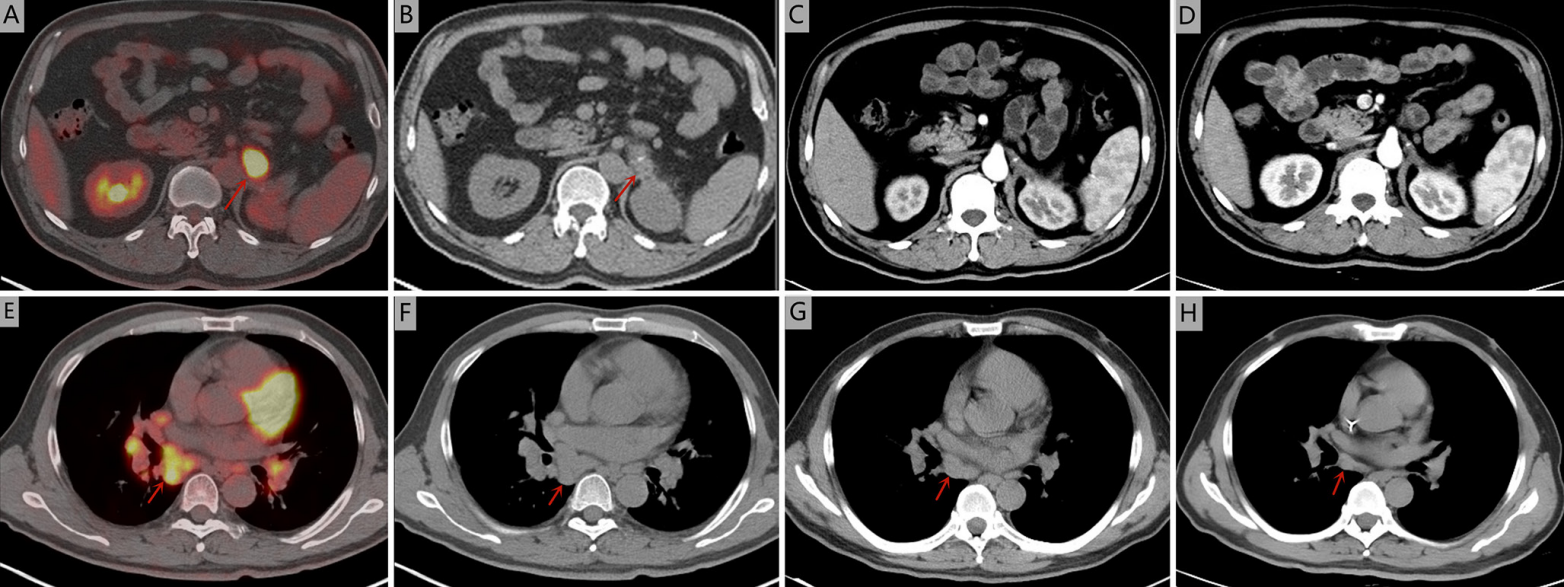
Seven months after operation, positron emission tomography/computed tomography was performed. **(A, B)** The soft tissue mass in the operative area was accompanied by an abnormal increase of fluorodeoxyglucose metabolism (maximum standardized uptake value, 21.0). **(E, F)** The larger lymph nodes were located in the 8R area (4.2× 1.9 cm, maximum standardized uptake value, 7.9). Computed tomography during treatment showed that the tumour lesions disappeared in the arterial phase and the mediastinal and mediastinal-hilar lymph nodes were significantly reduced or had disappeared. **(C, G)** after two cycles of chemotherapy. **(D, H)** after six cycles of chemotherapy.

A 51-year-old man was admitted to Zhejiang Hospital at the end of June 2020, complaining of lower back pain of over a month’s duration. He had a 10-year history of diabetes, controlled with oral metformin (2 tablets daily). After computed tomography (CT) examination, he was diagnosed with adrenal tumour and no abnormality in other parts. Due to the limitation of focus and the possibility of malignancy considered in imaging, a retroperitoneal laparoscopic resection of the left adrenal tumour was performed under general anaesthesia. During surgery, it was found that the tumour, identified in the left adrenal region, was about 4 cm in diameter, with a complete capsule and obvious adhesion to the left upper pole. Then, the tumour body and adrenal tissue were completely removed. Postoperative pathology revealed an adrenal angiogenic malignant tumour with massive necrosis (combined with morphology and immunohistochemistry, an epithelioid angiosarcoma was also considered), and a negative margin (R0 resection). (**Figures 1A, B**). The results of immunohistochemistry were as follows: CD34 (+), CD31 (+), ERG (partially weak+), FLI-1 (partially weak+), CK (+), CK7 (+), CK20 (–), CD56 (-), CgA (-), Syn (-), SMA (-), S-100 (-), desmin (-), HMB 45 (-), melan-A (-), and Ki67 (+, 20%) (**Figures 1C, D**). The patient decided not to undergo chemotherapy despite medical advice but followed up regularly after surgery. Seven months after operation, a positron emission tomography/computed tomography (PET-CT) examination was performed in response to further abdominal and back pain. The results showed a soft tissue mass in the operative area that was accompanied by an abnormal increase of fluorodeoxyglucose metabolism (maximum standardized uptake value, 21.0), and the diameter of the larger cross-section was about 2.4 × 1.6 cm. Furthermore, the lesion involved the left renal upper pole and left renal fascia, and the fat space around the lesion was slightly blurred (**Figures 2A, B**). Meanwhile, multiple enlarged lymph nodes were found in the bilateral clavicular area, bilateral mediastinal-hilar, mediastinum, bilateral anterior diaphragm, left retrocrural space, hepatic hilum, retroperitoneum (perirenal and pericaval) and left inguinal area, and the larger ones were located in the 8R area (4.2× 1.9 cm, maximum standardized uptake value, 7.9, **Figures 2E, F**). Postoperative recurrence of adrenal epithelioid angiosarcoma was comprehensively considered. The patient was then admitted to the Zhejiang Cancer Hospital and given 10 mg oxycontin every 12 h to control the pain. Given the extent of the local recurrence and possibility of new distant metastatic disease, our multi-disciplinary team discussions concluded that further surgery would not be indicated and that systemic chemotherapy should be considered. At the end of January 2021, the patient received combination chemotherapy, consisting of six cycles of liposomal doxorubicin (25 mg/m^2^ on day 1) plus paclitaxel (90 mg/m^2^ on days 1 and 8) once every 3 weeks. Fortunately, the patient had good tolerance and no obvious adverse reactions to the therapy (grade II peripheral neurotoxicity only, manifested as numbness of hands and feet). In addition, after two cycles of chemotherapy, the pain was relieved. At that time (after two cycles of chemotherapy), a CT scan showed a disappearance of the soft tissue tumour lesions in the operation area and a significant reduction or disappearance of the mediastinal and mediastinal-hilar lymph nodes (**Figures 2C, G**). He achieved partial response (PR) according to the RECIST 1.1 criteria. Further, he underwent four cycles of the treatment, and a total of six cycles ended in mid-May 2021 (day 1 of cycle 6). After six cycles of chemotherapy, a CT re-examination showed that the soft tissue tumour lesions in the operation area were responding to the treatment, and the diameters of the larger lymph nodes (8R) were less than 1 cm (**Figures 2D, H**). Finally, he had achieved a CR.

## Discussion

Adrenal gland angiosarcomas are very rare malignant tumours, most of which have been reported to have characteristic epithelioid morphology rather than spindled morphology (1, 3). Because of their rarity, their diagnosis may be challenging (3). Several differential diagnoses should be considered, including adrenal cortical carcinoma, pheochromocytoma, metastatic carcinoma, and metastatic melanoma. However, imaging findings of primary adrenal epithelioid angiosarcomas are classically non-specific and the cytology is considered to be exceedingly difficult for diagnosis. Thus, the most reliable and definitive diagnostic method is a combination of histology and immunohistochemistry.

The histologic features of epithelioid angiosarcoma include the presence of interlacing vascular spaces lined by endothelial cells that show nuclear pleomorphism, increased mitotic activity, abundant amphophilic or eosinophilic cytoplasm, round-to-irregular vesicular nuclei, and accentuated nucleoli (1). In addition, immunohistochemistry is of great value in the diagnosis of epithelioid angiosarcoma. Positive staining for ERG, CD34, and CD31, which are vascular endothelial markers, indicate that the tumour has endothelial characteristics (1, 6). FLI-1 is also used as an endothelial marker, but it is not specific and can be expressed in other soft tissue sarcomas. In the present case, the patient showed CD34 and CD31 positivity. At the same time, the ERG and FLI-1 were weakly positive. The above findings can be used to diagnose epithelial angiosarcomas, and melan-A negativity can serve as an ancillary tool in distinguishing them from adrenal cortical tumours (7).

According to previous reports, primary adrenal epithelioid angiosarcomas, presenting as non-functional masses (1), are associated with a male predilection and an age range of 60-70 years. Symptoms are non-specific, and the most common clinical feature is abdominal pain. In some cases there are no symptoms, in others, symptoms may range from significant weight loss, abdominal mass, episodic fevers, anorexia, and weakness (2). The patient presented with abdominal and back pain, which relieved after operation and after the tumour shrunk. Adrenal epithelioid angiosarcoma is a highly aggressive malignant tumour, in which the prognosis is generally poor, with a 5-year survival rate of approximately 20% (1, 2, 8). Advanced age, increased tumour size, high-grade, and a proliferative index >10% are considered adverse prognostic factors (1, 9). Because of its rarity, the treatment of this tumour has no uniform standard.

We searched databases (PubMed, Cochrane Library electronic databases, Google Scholar) for primary adrenal epithelioid angiosarcoma cases reported in the English literature over the past decade, and about 40 articles were found. However, when we carefully reviewed the information associated with these cases, only half of the reports covered the treatment of the disease. If the lesion was resectable, radical excision appeared to be justified (2, 10). Furthermore, doxorubicin-based chemotherapeutic regimens and adjuvant radiation therapy potentially resulted in a decreased risk of recurrence for localised resectable disease (2, 11). There were few reports on treatment for recurrent or advanced adrenal epithelioid angiosarcomas. Palliative therapy with cytoxan, doxorubicin, methotrexate, and vincristine were used, but the effect was unsatisfactory (12). A summary of previously reported cases which involved the treatment of recurrent or advanced adrenal epithelioid angiosarcoma are listed in **Table 1**.

**Table 1:** Summary of previously reported cases which involved the treatment of recurrent or advanced adrenal epithelioid angiosarcoma.

Reference	Sex	Age	Surgery	Adjuvant therapy	Recurrence time	Treatment of recurrence	Curative effect	Overall survival
Sarah Imran et al. (13)	F	38	None	None	None	PTX	NA	NA
Takizawa et al. (14)	M	66	Resection	None	3 months	PTX	Not controlled	died with another disease at 1.5 years
Kristine et al. (15)	F	53	Resection	None	NA	Chemotherapy+ Radiotherapy	Not controlled	died 3 months after metastasis
Grajales-Cruz et al. (16)	M	69	Resection	AI	1 year	Radiotherapy	Not controlled	died some weeks after recurrence
Sung et al. (17)	M	42	Resection	Radiotherapy	9 months	Resection	Not controlled	NA

NA, not available; M, male; F, female; AI, adriamycin/ifosfamide; PTX, paclitaxel.

The efficacy of liposomal doxorubicin in sarcomas was also generally limited, but it was considered to be an option for patients who could not tolerate more intensive chemotherapy (2). According to the guidelines for angiosarcoma, paclitaxel-based chemotherapy could be effective and was often used in a first- or second-line setting, but a study of 30 patients with angiosarcoma who received paclitaxel showed a response rate of 19% (2). We, therefore, questioned whether combination chemotherapy with liposomal doxorubicin and paclitaxel would be a more effective treatment. Several studies had shown that combination chemotherapy with doxorubicin and paclitaxel had a synergistic antitumour effect on patients with advanced solid tumours, such as breast cancer, ovarian cancer, and head and neck cancer (18, 19). Moreover, compared with doxorubicin, liposomal doxorubicin is not rapidly absorbed by the liver, circulates for prolonged periods in the bloodstream, accumulates in tissues with increased vascular permeability, such as tumour tissues, and has low cardiac toxicity (18, 20). A phase II study showed that the combination of liposomal doxorubicin and paclitaxel is a safe and modestly efficient first-line treatment in patients with advanced soft tissue sarcoma, with an overall response rate of 16% (21). We, therefore, used combination chemotherapy with liposomal doxorubicin and paclitaxel for this patient, with a successful result.

This study has certain limitations in that it remains unclear whether combination chemotherapy with liposomal doxorubicin and paclitaxel is suitable for all patients with adrenal epithelioid angiosarcomas, including angiosarcomas of other viscera. The accumulation of more cases will further reveal the effect of combination chemotherapy on this disease.

## Conclusion

In summary, we have presented a case of a 51-year-old male with a postoperative recurrence of adrenal epithelioid angiosarcoma. He underwent palliative chemotherapy combined with liposomal doxorubicin and paclitaxel. Fortunately, he obtained CR. This case report indicates that combination chemotherapy with liposomal doxorubicin and paclitaxel may be a preferred therapy for recurrent or advanced adrenal epithelioid angiosarcoma.

## Data Availability Statement

The original contributions presented in the study are included in the article/supplementary material. Further inquiries can be directed to the corresponding author.

## Ethics Statement

This study was approved by the Zhejiang Cancer Hospital Medicine Ethics Committee. The patient provided their written informed consent to participate in this study. Written informed consent was obtained from the individual for the publication of any potentially identifiable images or data included in this article.

## Author Contributions

HW and QZ acquired the data. JM analysed the radiological images. YW analysed the histological images. HW, JM, YW, and QZ prepared the manuscript. All authors contributed to the article and approved the submitted version.

## Conflict of Interest

The authors declare that the research was conducted in the absence of any commercial or financial relationships that could be construed as a potential conflict of interest.

## Publisher’s Note

All claims expressed in this article are solely those of the authors and do not necessarily represent those of their affiliated organizations, or those of the publisher, the editors and the reviewers. Any product that may be evaluated in this article, or claim that may be made by its manufacturer, is not guaranteed or endorsed by the publisher.
